# Measuring Self-Perceptions of Aging: Differences Between Measures When Predicting Health Outcomes

**DOI:** 10.1093/geroni/igaa057.1555

**Published:** 2020-12-16

**Authors:** Jordan Boeder, Dwight Tse

**Affiliations:** 1 Claremont Graduate University, Claremont, California, United States; 2 The Chinese University of Hong Kong, Hong Kong, China

## Abstract

The majority of self-perceptions of aging (SPA) research uses either a combination of the Age-related Cognition (AgeCog) scales of Ongoing Development and Physical Loss, or the Attitudes Towards Own Aging (ATOA) subscale to assess views on aging. Although these scales are used interchangeably, the valence (positive/negative) and the specificity of the view on aging (domain-based/general) being assessed are not consistent. This study investigates how different measures of SPA relate to one another and whether they differentially predict various types of health outcomes (psychological/physiological; well-being/ill-being). Data from the 2008 and 2014 waves of the German Aging Survey (DEAS; n=3,745), a population-based representative survey of adults aged 40 to 95, was used to examine the relationship between the AgeCog scales and the ATOA subscale, as well as the differences in the types of health outcomes each predicts. The correlations between the AgeCog scales and the ATOA were higher than the correlation between the AgeCog scales (p < .001). The AgeCog scale of Ongoing Development significantly predicted psychological health outcomes across a six-year period, while the AgeCog scale of Physical Loss and the ATOA subscale predicted both physiological and psychological health outcomes. Evidence supports using the AgeCog scale of Ongoing Development to predict domain-relevant, psychological health outcomes. However, the multidimensionality of SPA is best measured by the ATOA subscale or a combination of the two AgeCog scales. Both forms of measurement were found to maximize the amount of explained variance for psychological and physiological indicators of well-being and ill-being.

